# Postpyloric nutrition to prevent emergencies – a step away from repeat inpatient care in children with methylmalonic acidaemia and propionic acidaemia – a case report of four cases

**DOI:** 10.3389/fped.2023.1078425

**Published:** 2023-02-06

**Authors:** Stefan Schumann, Frank Risto Rommel, Serdar Cantez, Evdokia Alexanidou, Clemens Kamrath, Jan de Laffolie

**Affiliations:** Department of General Pediatrics and Neonatology, University of Giessen, Giessen, Germany

**Keywords:** postpyloric nutrition, J-PEG, prevention of emergencies, methylmalonic acidaemia, MMA, propionic acidaemia, PA, metabolic decompensation

## Abstract

Methylmalonic acidaemia (MMA) and propionic acidaemia (PA) are very rare autosomal recessive inherited metabolic diseases from the group of organoacidopathies. Katabolism due to minor infections can lead to metabolic decompensation including hyperammonemia and ketoacidosis, especially in small children. We present data from a small cohort to clarify whether placement of a percutaneous endoscopic gastrostomy with jejunal tube (J-PEG) reduce metabolic imbalances and hospital stays. The aim is to prevent emergencies from occurring by preventing metabolic derailments at an early stage. 4 patients with MMA (*N *= 3) or PA (*N *= 1) were included. Data were collected at every investigation, in particular pH value, pCO2, bicarbonate, base excess, ammonia and lactate. Due to repeated metabolic derailments, a percutaneous endoscopic gastrostomy was placed for postpyloric nutrition. In conclusion, placement of a percutaneous endoscopic gastrostomy with postpyloric tube appears to reduce the rate of metabolic decompensations. In addition, hospital stays and especially the number of treatment days can be reduced. This method, especially the placement of a postpyloric tube could enable parents to prevent catabolism when vomiting begins by continuously feeding through the jejunal part, as a step to prevent a metabolic emergency from occurring.

## Introduction

Methylmalonic acidaemia (MMA) and propionic acidaemia (PA) are very rare autosomal recessive inherited metabolic diseases from the group of organoacidopathies. Affected children suffer from poor feeding, vomiting and recurrent metabolic decompensations leading to coma and even death if not treated appropriately (1). Catabolism during episodes of fever, infection or vomiting lead to metabolic deterioration including metabolic acidosis and hyperammonemia (2, 3). An early reversal of a catabolic state is therefore essential. Cases of acute illnesses, e.g., early childhood infections, lead to frequent inpatient admissions of patients with MMA and PA, because monitoring and intravenous administration of fluids is mostly not feasible at home. Nutritional treatment by placement of a prepyloric percutaneous endoscopic gastrostomy (PEG) is described (4). However, the administration of food *via* the PEG does not reduce recurrent vomiting, which is a common problem in these patients.

**Figure 1:**
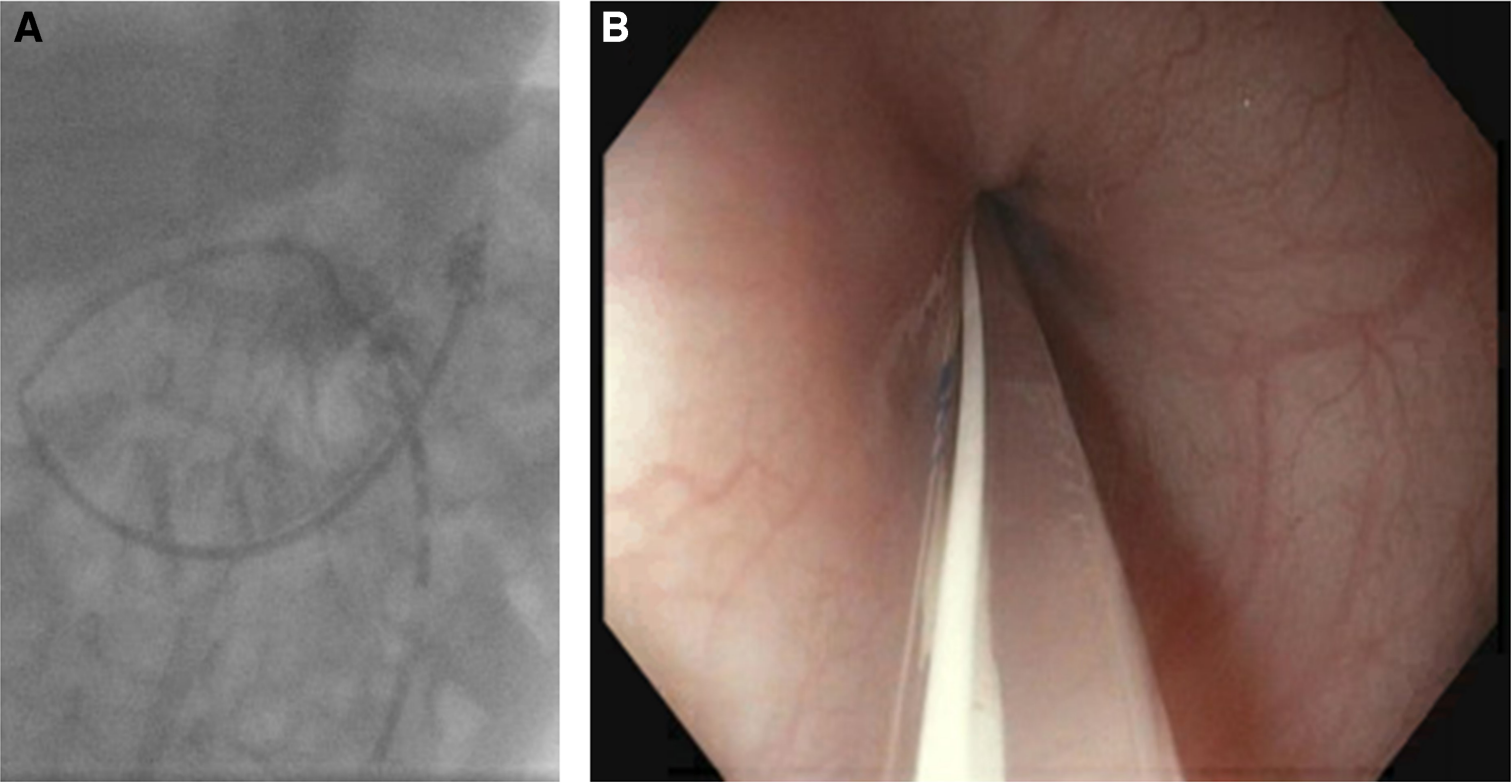
(**A**) A J-PEG is shown using an x-ray. (**B**) A J-PEG in front of the pylorus, recorded during endoscopic placement.

We present a small cohort of patients whose metabolic imbalances and hospital admissions due to catabolism were reduced after placement of a percutaneous endoscopic gastrostomy with jejunal tube (J-PEG) for postpyloric feeding in the home environment in case of (suspected) minor infections or vomiting (Figure 1) to reduce the risk of metabolic decompensation and emergencies.

## Methods

We retrospectively analyzed data of four patients with MMA (*N* = 3) and PA (*N* = 1) from October 2013 to November 2021 (Supplementary Table S1). Due to repeated metabolic derailments, a J-PEG was placed for postpyloric nutrition in every patient. Values of pH value, pCO2, bicarbonate, base excess, ammonia and lactate were analyzed at every clinical visit during this period. The interquartile range (IQR) for identifying outliers was calculated.

## Results

**Figure 2:**
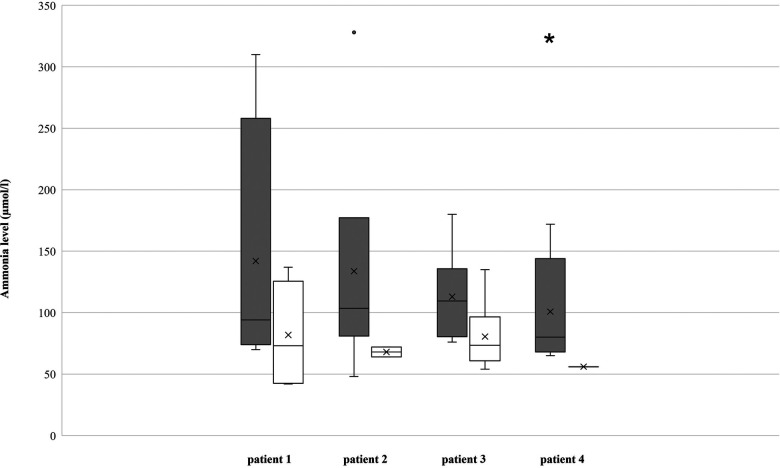
Ammonia levels of the patients, measured in µmol/L. *The maximum value of patient 4 with 975 µmol/L was left out, as a special outlier in a critical time of disease.

**Figure 3:**
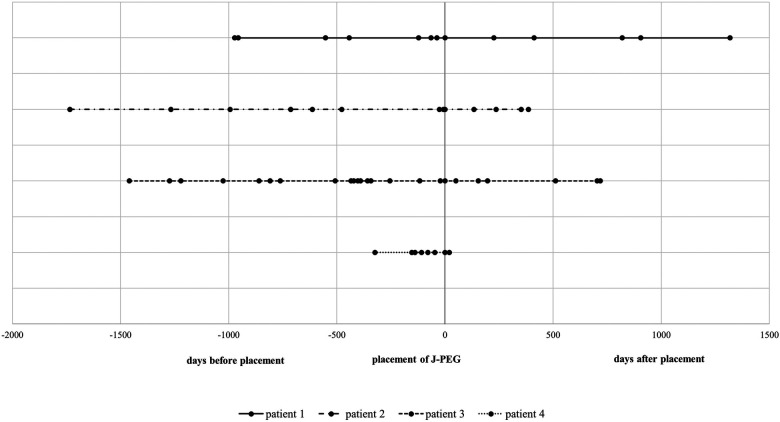
Hospital stays of patients. The vertical line shows the timing of J-PEG placement. Dots in lines represent hospital stays spaced from time of J-PEG placement.

In patient #1, the maximum ammonia level (Figure 2) before placement of J-PEG was 310 µmol/L (median 94, IQR 82 to 154). Minimum base excess was −16.4 mmol/L (median −8.3, IQR −12.7 to −4.6). After placement of J-PEG, the maximum ammonia level was 137 µmol/L (median 73, IQR 43 to 114) and minimum base excess was −13.9 mmol/L (median −8.6, IQR −11.3 to −5.9). The number of hospital admissions (Figure 3) was reduced from 3.0 per year with a median of treatment days of 4.5 (IQR 3.8 to 13.3) to a number of hospital days of 0.8 per year with a median of treatment days of 8.0 (IQR 6.5 to 9.5).

In patient #2, the maximum ammonia level before placement of the J-PEG was 328 µmol/L (median 104, IQR 92 to 124) and the minimum base excess was −21.7 mmol/L (median −13.4, IQR −16.2 to −5.2) (Figure 3). The number of hospital admissions per year was 1.9 with a median duration of 8.0 days (IQR: 3.8 to 14.0). After placement of the J-PEG, the maximum of ammonia level was 72 µmol/L (median 68, IQR 66 to 70) and the minimum base excess was −7.2 mmol/L (median −0.4, IQR −2.3 to 0.1). The yearly number of hospital admissions was 1.1 and the median duration was 5.0 days.

In patient #3, the maximum ammonia level before placement of the J-PEG was 371 µmol/L (median 110, IQR: 88 to 119), the minimum base excess was −25.2 mmol/L (median −5.8, IQR: −15 to −5.3) (Figure 2). After placement of the J-PEG, the maximum ammonia level was 135 µmol/L (median 74, IQR: 62 to 94) and minimum base excess was −13.3 mmol/L (median −9.2, IQR: −11.7 to −5.6). The number of metabolic derailments and subsequent hospital admissions did not decrease, however, the number of days in the clinic was reduced per admission (pre J-PEG: 3.1 hospital admissions per year with a median duration of 9.0 days (IQR: 8.0–12.3); post J-PEG: 2.8 hospital admissions per year with a median duration of 7.0 days (IQR: 5.0–9.0) (Figure 3). The patient developed a chronic kidney failure as a result of MMA, which also increased the number of metabolic decompensations (5).

Our currently youngest patient #4 was admitted 7.9 times with a median treatment duration of 8.0 days (IQR: 5.3 to 14.5) until the age of 11 months (Figure 3). The maximum ammonia level in this period was 975 µmol/L (median 98, IQR: 73.3–158) with a base excess of −29.2 mmol/L (median −29.2, IQR: 0) (Figure 2). Data after the placement of the J-PEG are not yet available. (Supplementary Table S2: Results of our cohort).

## Discussion

Metabolic decompensation in patients with MMA and PA lead to intoxication with organic acids. High levels of ammonia are associated with acute encephalopathy, seizures, movement disorders and stroke-like episodes (3). Early prevention should therefore be the first step in a series of emergency treatment options. In patients receiving a J-PEG, parents can use the jejunal tube of the J-PEG for maintenance enteral nutrition during episodes of vomiting to prevent catabolism and the accumulation of toxic metabolites. In our case series, the placement of a percutaneous endoscopic gastrostomy with a postpyloric tube (J-PEG) appears to reduce the rate of metabolic decompensations. One reason is the avoidance of vomiting by postpyloric administration of food with the aim of preventing catabolism.

In addition, our cohort shows that the rate of hospital admissions and especially the number of treatment days in the clinics could be reduced. Parents can use postpyloric feeding at home as a first step. It is conceivable that this can lead to a reduced number of presentations in the emergency room and to a reduced alerting of the emergency services. In the case of hospital treatment, patients can be discharged more quickly for outpatient treatment.

Further studies are needed to follow-up and to compare the rate of metabolic decompensations with patients without the placement of a postpyloric tube.

## Conclusion

In conclusion, the presented approach could enable parents to prevent catabolism when vomiting begins by continuously feeding through the jejunal tube. An example of an emergency plan can be, to change the diet to low-protein food *via* a feeding pump in the case of illness (vomiting, fever) and to give it *via* the jejunal tube in the case of vomiting. The procedure described can be an emergency plan. However, patients are often fed jejunally for a long period of time. Vomiting and the associated retroperistalsis can lead to a displacement of the jejunal tube, which then has to be corrected, for example with a guide wire with imaging and in rare cases also endoscopically. These aspects were not examined in the present case series, but should be included in further research.

## Data Availability

The original contributions presented in the study are included in the article/**Supplementary Material**, further inquiries can be directed to the corresponding author.
