# Supplementary material for: Postpyloric nutrition to prevent emergencies – a step away from repeat inpatient care in children with methylmalonic acidaemia and propionic acidaemia – a case report of four cases
Source: Front Pediatr. 2023 Feb 6;11:1078425. doi: 10.3389/fped.2023.1078425 (PMC9939511; doi:10.3389/fped.2023.1078425)
Supplement: Supplementary file 1 [file Table1.pdf]

| | type of mutation |
|---|---|
| | |
| patient 1 | methylmalonic acidaemia,<br>compound heterozygous mutation in the MMAB gene (c.557G>A/Del),<br>encoding the cobalamin adenosyl transferase |
| patient 2 | methylmalonic acidaemia,<br>no vitamin B12 responsiveness,<br>homozygous methylmalonyl-CoA mutase mutation (mut 0) |
| patient 3 | methylmalonic acidaemia,<br>compound heterozygous mutation in the MUT gene |
| patient 4 | propionic acidaemia,<br>homozygous mutation c.415C>T ; p.Gln139Ter in the PCCB gene |

**Table 1:** Type of mutations of our patients.
