# Supplementary material for: Postpyloric nutrition to prevent emergencies – a step away from repeat inpatient care in children with methylmalonic acidaemia and propionic acidaemia – a case report of four cases
Source: Front Pediatr. 2023 Feb 6;11:1078425. doi: 10.3389/fped.2023.1078425 (PMC9939511; doi:10.3389/fped.2023.1078425)
Supplement: Supplementary file 2 [file Table2.pdf]

		maximum ammonia level (µmol/l)		minimum base excess (mmol/l)		number of hospital admissions (per year)		treatment days (days)	
		pre	post	pre	post	pre	post	pre	post
patient 1		310	137	-16.4	-13.9	3.0	0.8		
	median	94	73	-8.3	-8.6			4.5	8.0
	IQR	82 to 154	43 to 114	-12.7 to -4.6	-11.3 to -5.9			3.8 to 13.3	6.5 to 9.5
patient 2		328	72	-21.7	-7.2	1.9	1.1		
	median	104	68	-13.4	-0.4			8.0	5.0
	IQR	92 to 124	66 to 70	-16.2 to -5.2	-2.3 to 0.1			3.8 to 14.0	
patient 3		371	135	-25.2	-13.3	3.1	2.8		
	median	110	74	-5.8	-9.2			9.0	7.0
	IQR	88 to 119	62 to 94	-15 to -5.3	-11.7 to -5.6			8.0 to 12.3	5.0 to 9.0
patient 4		975	data not yet available	-29.2	data not yet available	7.9	data not yet available		data not yet available
	median	98		-29.2				8.0	
	IQR	73.3 to 158		0				5.3 to 14.5	

Table 2. Results of our cohort.
